# That Is Sound Which Works

**Published:** 1918-01-05

**Authors:** 


### "THAT IS SOUND WHICH WORKS."

The efficiency of the new broom is proverbial and its advent invariably stimulates great expectations. Even a record of disappointments following kindred experiences does not lessen the welcome which attends the newcomer. The failures were themselves no doubt once new. But they are old now, and it is the new which excites hope to spring eternal in the human breast. On a personal and domestic plane such experiences are common, but the present conjunction of events has made us familiar with similar happenings in high places. Even such dignified organisations as the Admiralty and the Ministry of National Service seem to suffer the ordinary human lot. From an announcement in the lay press we learn that this latter organisation has at last secured the opinion and direction of "the soundest of modern investigators," and that by the light of the "new medicine" the recruiting of the Army is to be guided to success. In terms of this order the public is invited to confide in the new arrivals. The old management, with its effete methods and its blunders, is happily removed, and the newest light illuminates the men now in possession.

The authority which tells us of these events—accomplished and to come—also informs us that the "new medicine" takes for its basis the phrase which we place at the head of this article. "That is sound which works" is the principle which is to rule the acceptance or rejection of the recruit, and the art of the physician must be content to occupy a secondary place. What a man can do is to be one of the chief tests of his fitness or unfitness for military service, and in these circumstances a medical examination would seem to be largely superfluous. For "what a man can do" will appear from the record of his occupation, or can be determined by a ready scheme of exercises. Why, then, trouble with the delicacies of the stethoscope or other niceties of clinical investigation? The man's performance is its own testimony, and with this the "new medicine" is apparently fully content.

For our own part we had thought that the medical art was capable of determining something which could not be learned from an individual's judgment on his own sensations; that it was able to appreciate facts which produce no subjective experiences; and that it had some ability to estimate the effects of future stresses and other allied conditions. Similarly, we believed that even the "old medicine" was not blind to the considerations which are now claimed as the special virtue of its successor. Perhaps its voice was not so much heard in the streets, but this we were always disposed to place to its credit; and, here at all events, we think the "new" a less attractive proposal. When the lay press embarks on the technicalities of medicine it generally makes a poor exhibition, and its ignorance leads to an emphasis which to the instructed is ridiculous enough. It may well be that the latest excursion is a further illustration of this experience, and we are willing to leave it at that. But nowadays we hardly wonder "how these things get into the papers" and the present inspiration is hardly a happy one. The public is prepared for some startling new departure. But we fancy that experience will show little that is new beyond the gentle art of self-advertisement.

